# Gamma-irradiated fowl cholera vaccines formulated with different adjuvants induced antibody response and cytokine expression in chickens

**DOI:** 10.3389/fimmu.2025.1513443

**Published:** 2025-02-27

**Authors:** Eyerusalem Belay, Molalegne Bitew, Saddam Mohammed Ibrahim, Bereket Dessalegn, Solomon Lulie Abey, Haileyesus Dejene, Mastewal Birhan, Dawit Duffera, Eyob Asefa, Liyuwork Tesfaw, Takele Abayneh, Kedir Sherefa, Wubet W/Medhin, Yeneneh Tesfaye, Keyru Tuki, Esayas Gelaye, Richard Thiga Kangethe, Viskam Wijewardana, Carla Bravo De Rueda

**Affiliations:** ^1^ College of Veterinary Medicine and Animal Sciences, University of Gondar, Gondar, Ethiopia; ^2^ Health Biotechnology Directorate, Bio and Emerging Technology Institute (BETin), Addis Ababa, Ethiopia; ^3^ Vaccine Research and Development Directorate, National Veterinary Institute, Debre Zeit, Ethiopia; ^4^ Food and Agriculture Organization (FAO) of the United Nations, Emergency Centre for Transboundary Animal Diseases (ECTAD), Addis Ababa, Ethiopia; ^5^ Animal Production and Health Section, Joint FAO/IAEA Centre of Nuclear Techniques in Food and Agriculture, Department of Nuclear Sciences and Applications, International Atomic Energy Agency, Vienna, Austria

**Keywords:** adjuvants, chicken, cytokines, fowl cholera, gamma-irradiated vaccines, humoral immunity, mucosal immunity, *P. multocida*

## Abstract

Fowl cholera is one of the most serious and economically important infectious diseases of poultry caused by *Pasteurella multocida*. Formalin-inactivated vaccine, administered intramuscularly, is widely used in Ethiopia with a low success rate. Gamma irradiation is an effective approach to inactivate pathogens for vaccine development. In a previous study, we reported the feasibility of developing gamma-irradiated vaccines that induced both systemic and mucosal antibody responses with complete protection against homologous lethal challenge. In the present study, we aimed to broaden our understanding of the immunogenicity of the gamma-irradiated vaccines by including peripheral blood mononuclear cells (PBMC) response analysis. A total of 156 eight-week-old fowl cholera-specific antibody negative Bovans Brown chickens were utilized in this experiment. The performances of gamma-irradiated *P. multocida* vaccines formulated with different adjuvants, Montanide Gel 01 PR (G-1), Carbigen^®^ (G-2), Emulsigen-D^®^+aluminum hydroxide gel (G-3), and Emulsigen-p^®^ (G-4) were evaluated in comparison with the formalin-inactivated vaccine (G-5) and unvaccinated control (G-6). Chickens received two doses of the vaccines at days 0 and 21. Sera, tracheal, and crop lavage were collected at days 0, 21, 35, and 56 to assess IgG and IgA levels using indirect and sandwich ELISA, respectively. PBMC proliferation was compared between vaccinated and unvaccinated controls. In addition, vaccination-induced expression of cytokine genes was analyzed in PBMC using qPCR. Chickens were challenged with 2.5x10^7^ CFU/ml of *P. multocida* biotype A intramuscularly one day after day-56 sampling. Significant serum IgG titers were detected three weeks after primary vaccination in G1, G3, and G5. IgG titer substantially increased in all vaccinated groups two weeks post-booster dose. IgA response was induced by gamma-irradiated vaccines but not formalin-inactivated vaccines. Only PBMC from vaccinated chickens proliferated in response to re-stimulation with *P. multocida* antigen, indicating vaccine-specific priming. Interestingly, gamma-irradiated vaccines resulted in a higher fold change in mRNA transcripts of IFN-γ (>1000-fold change) IL-6 (>500-fold change), and IL-12p40 (>200-fold change), which are hallmarks of a Th1 dominant response, which is essential to combat intracellular infection. Lastly, the candidate vaccines demonstrated various levels of protection, with Emulsigen-D^®^ containing vaccine rendering complete protection against homologous lethal challenge. In conclusion, gamma-irradiated vaccines can induce broad immune responses, humoral and cellular, and protect against severe outcome of fowl cholera. Therefore, this study has contributed to growing knowledge on the immunogenicity and efficacy of gamma-irradiated vaccines and has shown the potential of such a vaccine platform for field application in extensive as well as intensive farm settings.

## Introduction

Fowl cholera, caused by *Pasteurella multocida* is one of the serious infectious diseases of poultry (1). According to Molalegne et al. (2), the disease is endemic throughout the majority of Ethiopia and causes severe economic losses due to decreased productivity and mortality. Vaccines are one of the effective ways to control an outbreak within a flock (3) and both live attenuated and killed vaccines against fowl cholera are now available on the market (4, 5). Compared to live-attenuated vaccines, inactivated vaccines often have a greater safety profile as risk of reversion is avoided or significantly low. Additionally, they are less reactogenic, but they also have a lower immunogenicity and need multiple doses to produce a protective effect (6).

The development of vaccines using locally circulating strains is essential and desirable (7). For the pathogen to effectively elicit an immune response, its structure needs to be properly conserved (8). A variety of pathogen inactivation techniques such as gamma irradiation (8–10), chemical treatment (11), and heat inactivation (12) are available for use. Chemical inactivation using formalin is a common practice in the vaccine industry. However, this could cause alteration or damage to the surface antigenic structures thus affecting immunogenicity and efficacy. Furthermore, immunization with formalin-inactivated respiratory syncytial virus and measles vaccines had resulted in disease enhancement that was ascribed to the low avidity non-protective antibodies elicited due to formalin treated antigens (13). The other hallmark of formalin-killed vaccines is a Th-2 immunity which is implicated in vaccine associated pathologies (14). Similarly, even if effective in inactivating pathogens, heat inactivation is also reported to distort epitopes and induce less antibody titer than formalin-treated vaccines (15). Another study by Hashizume-Takizawa found that heat killed recombinant Salmonella enterica serovar Typhimurium could elicit neither systemic IgG nor mucosal IgA (16). Moreover, heat inactivation has been reported to yield inconsistent results (17).

Gamma irradiation technique uses ionizing radiation to specifically target nucleic acids while preserving surface antigenic protein, making it preferable to develop safe and immunogenic vaccines (18). It is also a convenient method as it precludes the need to remove chemical agent post-inactivation.

According to studies, gamma-irradiated vaccines possess better efficacy and shelf life when compared to live-attenuated and formalin-killed vaccines (9, 19, 20). In addition, vaccines developed by irradiation have been tested and reported as strong inducers of mucosal and humoral immune response (21, 22).

Mucosal vaccination is an effective and efficient method of immunization against mucosal pathogens as they are convenient to administer for large-scale campaigns and farm settings (23), and can induce long-lasting humoral and cellular immunity (24). Previously, we reported the feasibility of developing gamma-irradiated vaccines that induced both systemic and mucosal antibody responses with complete protection against homologous lethal challenge (9). In the current study, we aimed to broaden our understanding of the immunogenicity of the gamma-irradiated vaccines by including peripheral blood mononuclear cells (PBMC) response analysis. Cytokines are an integral part of the immune response in avian species of infection (25). They are involved in both inflammatory and specific immune responses to invasive microbes, which were evolved to protect the host from pathogens (26). As regulators of the initiation and maintenance of host defenses, cytokines ultimately determine the type of response generated and the effector mechanisms generated to mediate resistance (27). Thus, in addition to serum IgG and mucosal IgA, we assessed PBMC proliferation and the expression of a range of cytokine genes—IL-1β, IL-6, IL-12, IFN-γ, IL-4, and IL-22—that modulate the immune response to infection and vaccination (28), in response to gamma-irradiated and formalin-inactivated vaccines.

## Materials and methods

### Experimental site

This study was carried out from December 2022 to November 2023 at the National Veterinary Institute (NVI) in Bishoftu; the National Institute for Control and Eradication of Tsetse and Trypanosome (NICETT) in Addis Ababa; and the Bio and Emerging Technology Institute (BETin) in Addis Ababa.

### Sample size and experimental chickens

G*power provided a samples size of 149 using the following parameters—effect size: 0.3, power: 0.8, number of experimental groups: 6, and numerator df: 5. Thus, A total of 156 eight-week-old Bovans Brown chicks that were Fowl cholera (FC)-specific antibody negative (SAN), hatched from fertile eggs obtained from the National Veterinary Institute (NVI) were used in this experiment up to the age of eight weeks. In addition, the parental stock had no history of vaccination against FC. The experimental chicks were reared under strict farm biosecurity measures. Before introducing the chicks, the room was cover with wood shavings and formalin-fumigated and ventilated for three days. Throughout the experiment, the chickens had free access to food and water *ad-libitum*.

### Preparation of vaccine and challenge bacteria

Inoculum of *P. multocida* used in the vaccine formulation and challenge study was prepared according to the NVI’s standard operating procedure (29). Briefly, lyophilized avian *P. multocida* biotype A master cell bank obtained from the NVI (MK802880) was thawed, diluted with 2 ml tryptose soya broth (TSB), inoculated into sterile tryptose soya agar (TSA) supplemented with 10% horse serum, and incubated overnight at 37°C. A single colony was then taken into 2 ml TSB supplemented with 10% horse serum, and incubated for 7 h at 37°C. Next, 0.5 ml of the culture was transferred into 30 ml of TSB supplemented with 10% horse serum and incubated overnight at 37°C. Culture was up scaled by inoculating 300 ml of *P. multocida* biotype A production media with 7 ml of the overnight culture and incubating for 24 h at 37°C and 80 rpm. The culture was adjusted to 3.7x10^8^ CFU/ml using streak plate method of serial diluted culture and was ready to be used in the preparation of the vaccines.

Challenge bacterium was obtained after taking a pure TSA colony into 200 ml TSB and incubating for 7 h at 37°C. Adjustment was made so that each chicken received about 2.5x10^7^ CFU/ml of inoculum when evaluating protective efficacy of the vaccines.

### Molecular characterization of *P. multocida*

The master seed obtained from NVI was characterized microbiologically, biochemically, and molecularly to confirm its identity and purity. Similarly, the bacteria re-isolated from infection-challenged chicken were also confirmed molecularly.

Master seed was cultured in TSA overnight at 37°C. Swab samples from liver, heart, and spleen were collected in PBS, incubated in TSA supplemented with 10% serum at 37°C for 18 h, and stored in a freezer until the next step. The genomic DNA was extracted using DNeasy^®^ Blood and Tissue Kit (Qiagen, German town, MD, USA) as per the manufacturer’s recommendation. DNA was kept at −20°C awaiting PCR analysis. The capsular biosynthesis gene (capA), a 1044 bp gene, was amplified using the following primers: F: 5′-TGCCAAAATCGCAGTCAG-3′ and R: 5′-TTGCCATCATTGTCAGTG-3′.

All the PCR reactions were carried out in a final reaction volume of 25 µL comprising 12.5μl of 2xPCR master mix (Promega, USA), 2μl DNA template, 1μl of 10pmol of each primer, and 8.5 μL of dH2O. The PCR reaction consisted of an initial denaturation at 95°C for 5 min, followed by 35 cycles of reaction involving denaturation at 95°C for 30 s, annealing at 55°C for 30 s, and extension at 72°C for 30 s with a final extension at 72°C for 5 min. As negative control, DNA sample from *P. multocida* capsular serogroup B was used. Gel electrophoresis of the PCR products was done using 2.0% (w/v) agarose gel. After electrophoresis, the DNA was stained for 10 min in Ethidium bromide (0.5 μg/mL) and visualized using a UV trans-illuminator (Alpha imager, Germany).

### Gamma irradiation for inactivation avian *P. multocida*

The radiation experiment took place at the NICETT Radiation Laboratory in Addis Ababa, Ethiopia. The culture containing the target bacterial titer (3.7 x 10^8^ CFU/ml) was aliquoted into test tubes and spun at 4,000 x *g* at 4°C for 20 min. Then, the pellet was washed twice with PBS and resuspended in 20% trehalose. The bacterial cell pellet was subjected to gamma irradiation for a varying amount of time depending on the doses, ranging from 0.8 to 1.3 kGy, using a cobalt 60 irradiation machine (MDS Nordion, Canada) at a dose rate of 1.56 kGy/hr. The temperature range of the gamma chamber was maintained at 37–40°C. After completion of the irradiation process, each tube was carefully taken out of the gamma chamber and immediately stored at 4°C until further use. A non-irradiated culture was used as a control. The inactivation capacity of the different radiation doses was evaluated by subculturing serial dilutions of treated culture on TSA plates and estimating the CFU/ml.

### Formulation of gamma irradiated vaccines

Previously, the 1 kGy gamma-irradiated avian *P. multocida* vaccine was shown to be immunogenic and efficacious in chickens (9). Thus, it was selected for the vaccine preparation in this study. The avian *P. multocida* inoculum was prepared at a dose of 3.7 x 108 CFU/ml. Four different vaccines were formulated by mixing the bacterial inoculum with four different adjuvants. The adjuvant’s concentration varied according to the suppliers’ instructions: 20% for Montanide/01 PR gel, 15% for Emulsigen^®^-P, 6% for Carbigen^®^, and 15% for the combination of Emulsigen^®^-D and Alum. These adjuvants have been documented to be safe and enhance immunogenicity and efficacy of various experimental and licensed vaccines (9, 30, 31).

The sterility and purity of formulated vaccines were assessed using Gram’s staining and culturing on sterility test media including Sabouraud dextrose agar, TSA, and TSB.

### Experimental design

#### Vaccination, samples, and sampling schedule

Chickens were divided into six groups (G1 to G6) of 26 chickens each, based on the vaccine type they received, as follows: G1: vaccine adjuvanted with Montanide/01 PR gel intranasally (IN) at a dose of 0.3 mL; G2: vaccine adjuvanted with Carbigen^®^ IN at a dose of 0.5 mL; G3: vaccine adjuvanted with Emulsigen-D and Alum intramuscularly (IM) at a dose of 0.5 mL; G4: vaccine adjuvanted with Emulsigen^®^-P IM at a dose of 0.5 mL; G5: formalin-inactivated vaccine IM at a dose of 0.5 mL; and G6 was used as an unvaccinated control. A booster dose was administered 3 weeks after the initial dose.

**Figure 1:**
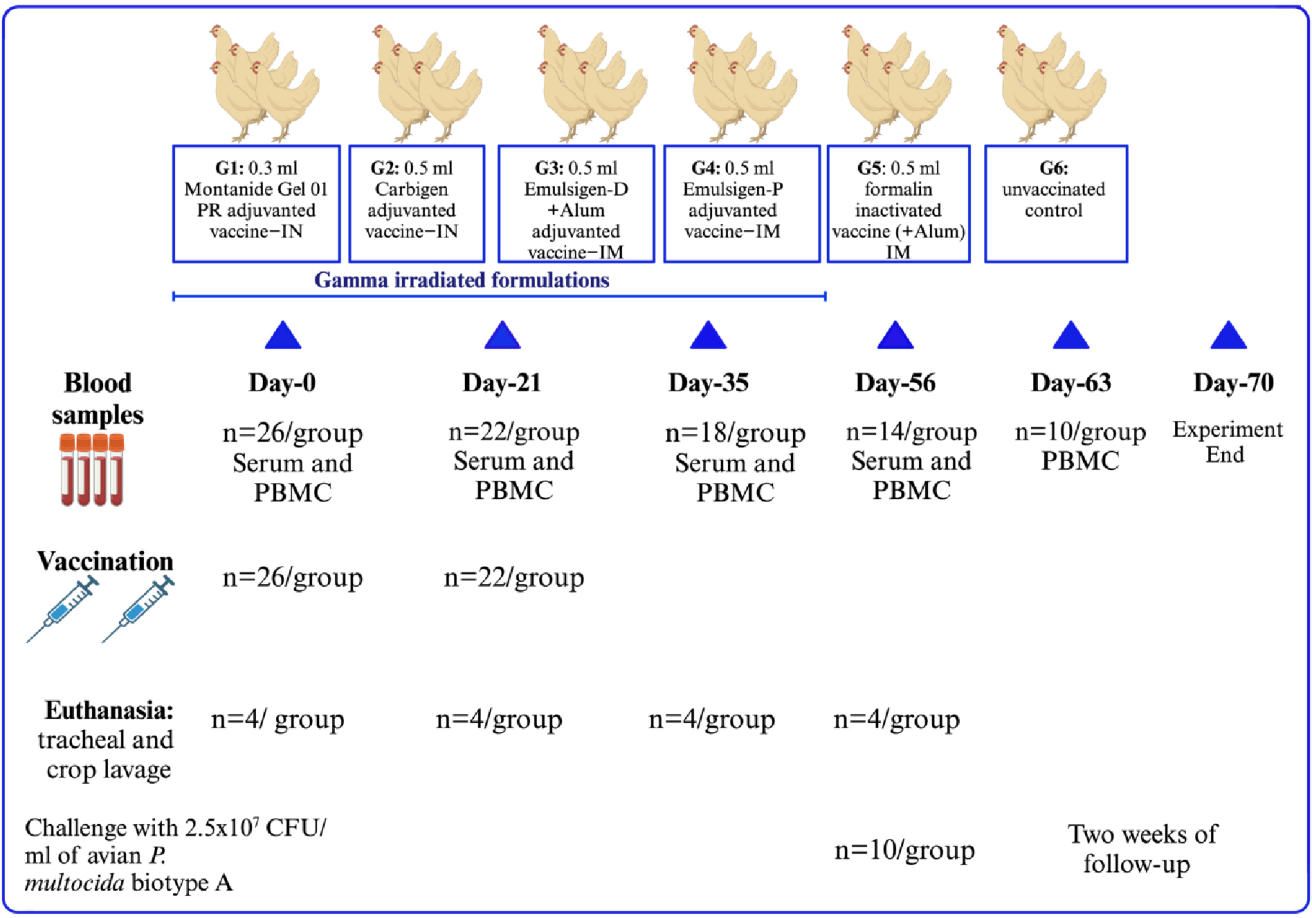
Experimental design of the study. A total of 156 chickens were classified into six groups depending on the type of vaccine administered. Individual chickens received 2 doses of their respective vaccines 3 weeks apart. Blood was sampled at days 0, 21, 35, and 56 for PBMC and serum analysis. In addition, seven days after the challenge, blood was obtained for cytokine gene expression analysis. Tracheal and crop lavage were also collected by repeatedly euthanizing four chickens. Ten chickens from each group were challenged with a lethal dose of avian *P. multocida* intramuscularly to assess the efficacy of the vaccines.

Blood samples were collected prior to vaccination and at days 21, 35, and 56 post-primary vaccination. Serum and PBMC were separated for antibody and cellular immune response analysis, respectively. Four chickens per group were euthanized according to the indicated schedule to collect tracheal and crop lavage to study mucosal immunity. The remaining 10 chickens per group were challenged to assess vaccine efficacy (**Figure 1**).

#### Safety assessment of the vaccines

Safety of the candidate vaccines was evaluated according to the harmonized requirements in VICH GL44 (32) which is endorsed by the World Organization for Animal Health (WOAH). Experimental chickens were monitored for adverse reactions daily for the entire period of the trial since the time of vaccination.

#### Serum and mucosal antibody response

Serum antibody response was assessed by quantifying IgG titer using a commercial indirect ELISA test kit (IDvet, France). Secretory IgA response was evaluated in tracheal and crop lavages using Sandwich ELISA (Chicken IgA ELISA Kit ab157691, Mybiosource, San Diego, USA). Optical density measurements were taken at 450nm.

#### Enrichment of PBMCs and their *in vitro* stimulation

Individual blood samples collected in Na–citrate tubes (Greiner Bio-One, Kremsmünster, Austria) were pooled as per their groups. Pooled blood samples were diluted in PBS containing 2 mM EDTA at a ratio of 1:2. Then, 3% dextran solution was added at a ratio of 1:0.4 and centrifuged at 50 x g for 20 min. The upper phase containing PBMCs was carefully layered onto 50 ml conical tubes containing Ficoll (Greiner Bio-One, Kremsmünster, Austria) (1:2) and centrifuged (Remi Lab World, Mumbai, India) at 800 x g for 35 min at 20°C with the brake off. After centrifugation, PBMC was harvested from the interphase between the bottom Ficoll and the upper plasma. Next, the PBMCs were transferred into 50 ml conical tube and washed twice by centrifuging at 1500 rpm for 7 min at 20°C using PBS. The resulting supernatant was decanted, and the pellet was reconstituted using 5 ml of RPMI-1640 media (UK). Then, cells were counted using an automated cell counter (EVETM, NanoEnTek) after mixing 10µL of cell suspension with 10µL of a 0.4% trypan blue solution.

The PBMCs, at a density of about 5×10^7^ cells/ml, were cultured overnight in RPMI supplemented with 10% fetal calf serum (Gibco™, Thermo Fisher Scientific), 5% chicken serum (Gibco™, Thermo Fisher Scientific), 100 U/mL penicillin, and 100 g/mL streptomycin at 37°C and 5% CO_2_ in untreated flat-bottomed 24-well culture plates (Eppendorf, Hamburg, Germany). To analyze vaccination induced expansion and activation of antigen specific immune cells, PBMCs were treated with gamma irradiated *P. multocida* antigen at a ratio of 1:1. Lymphocyte activation cocktail, a mixture of PMA (20–50 ng/ml) and ionomycin (0.5–1 g/ml), was added and used as a positive control. Cells were counted using hemocytometer. Following the overnight incubation, non-adherent cells were removed by washing the monolayers with sterile PBS.

#### RNA extraction and cytokine gene analysis using RT qPCR

RNA extraction from PBMCs was performed using Direct-zol™ RNA MiniPrep kit (Cat #: R2052) as per the manufacturer’s instructions. RNA concentration was determined spectrophotometrically (Biophotometer Plus, Eppendorf) at 260 nm, and isolated RNA was kept at -80°C until the next step. Then, cDNA was synthesized from 1µg of total RNA using a cDNA synthesis kit (Cat # K1612, Fermentas). Random hexamers were used to generate 15 µl of cDNA for every gene, and concentration was adjusted to 100 ng/µl for RT qPCR.

The genes for the following cytokines: IFN-γ, IL-1β, IL-4, IL-6, IL-12P40, and IL-22 were amplified using the primers indicated in **Table 1**. GAPDH was used as a reference gene. RT-qPCR was performed using SYBR^®^ Green Supermix (Cat # 1708882, USA) in a real-time thermocycler (Mastercycler^®^ ep realplex, model # 22331) using 7500 Real Time PCR System (Applied Biosystems). PCR conditions were the same for each targeted gene and are as follows: 50°C for 1 min, 95°C for 5 min, followed by 45 cycles of 95°C for 15 seconds and 58°C for 32 seconds. Cycling was terminated after 45 cycles with 95°C for 15 seconds, 60°C for 1 min, and 95°C for 15 seconds. Dissociation curves of the products were generated by increasing the temperature of samples incrementally from 55 to 100°C as the final step of the real-time PCR. Then, melting-curve analysis of amplified products was performed (**Table 1**).

**Table 1:** Sequences of the oligonucleotide primers used in the RT-qPCR to amplify cytokine genes of interest.

Target cytokines	Primer sequences		Size (bp)	Tm (°C)	Ref
IFN-γ	Forward	TGGCGTGAAGAAGGTGAAAGA	152	83.5	(25)
	Reverse	TCTGAGACTGGCTCCTTTTCT			
IL-1β	Forward	CTGAGTCATGCATCGTTTATGTTTC	120	76	(26)
	Reverse	AAATACCTCCACCCCGACAAG			
IL-4	Forward	TGAATGACATCCAGGGAGAGGTTT	179	82.5	(27)
	Reverse	ATTCAGGAGCTGACGCATGTT			
IL-6	Forward	GCTTCGACGAGGAGAAATGC	139	83	(25)
	Reverse	GCCAGGTGCTTTGTGCTGTA			
IL-12p40	Forward	AAAGACTGGGCCAAAAGACAAG	85	81.5	(27)
	Reverse	GCAAAGCGTGGACCACTCA			
IL-22	Forward	TGTTGTTGCTGTTTCCCTCTTC	60	79	(28)
	Reverse	CACCCCTGTCCCTTTTGGA			
GAPDH*	Forward	AGGGTGGTGCTAAGCGTGTT	142	83	(26)
	Reverse	AAGGGTGCCAGGCAGTTG			

*Reference gene.

#### Efficacy of the candidate vaccines

Ten chickens from each group were challenged intranasally with 2.5x10^7^ CFU/ml of avian *P. multocida* biotype A one day after the last sampling and were followed for 14 days. Necropsy and bacterial isolation were performed on chickens that were found dead. Gross lesions were recorded, and tissue samples were taken from lungs, livers, and spleens for bacterial isolation using TSA with 10% serum. Identification was then performed using morphology, Gram staining, and PCR.

### Data analysis

GraphPad Prism 8.4.3 (San Diego, California) was used to perform the statistical analysis and generate the graphs. Serum antibody titer was compared between different sampling time points within each group and across other groups using Friedman and Kruskal-Wallis tests respectively. Dunn’s *post-hoc* test was used when a difference between groups existed. One sample from day-35 of G3 was excluded from the dataset for being an outlier (significantly higher than the average). Both intragroup and intergroup comparisons of mucosal antibody response were performed using Kruskal-Willis test followed by Dunn’s test. The difference in the proliferative response of PBMCs from different groups was analyzed similarly,

Cytokine gene expression analysis was performed using the Livak’s method (2^-ΔΔ^ *^CT^*) for relative gene expression analysis (33). Target gene expression was normalized to the endogenous control, GAPDH. Relative fold change was determined by dividing the expression ratio of each target gene by their expression ratio in the control samples. The survival of chickens after infection challenge was analyzed using Kaplan-Meier curve analysis. The data were presented as mean ± standard error of the mean (SEM). Statistical significance was set at *p*<0.05.

### Ethical consideration

The experimental protocol was approved by the Animal Research Ethics Committee of the NVI (reference number: NVI/1453). All chickens were handled and euthanized humanely according to the ethical standards set in the international guiding principles for animal experiment research (34).

## Results

### Vaccine safety

All vaccinated chickens were followed up until challenge and we did not observe any abnormality in both the formalin and gamma-irradiated groups.

### Systemic IgG response

**Figure 2:**
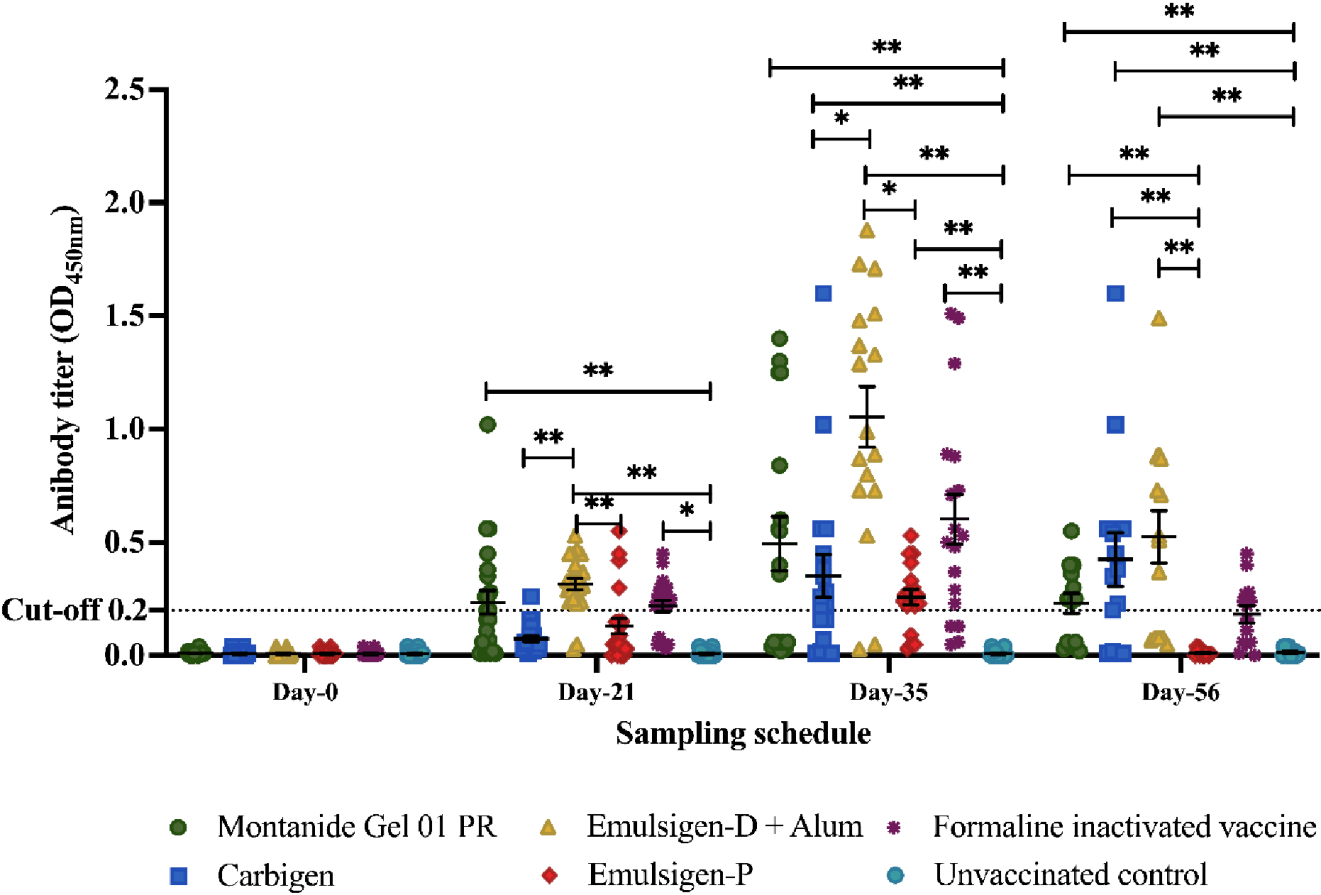
Serum IgG response to the candidate vaccines at days 0, 21, 35, and 56. Serum IgG titer was analyzed using indirect ELISA at 450nm. The mean ± SEM is shown for each group on sampling day. A cut-off value of 0.2 was used according to the manufacturer's instructions. Single (*) and double asterisk (**) represent *p*<0.05, Cohen's effect size value (*d* = .62) and p<0.01 (*d* = .72), respectively.

Significant serum IgG titer was detected three weeks after primary vaccination in G1, G3, and G5. IgG titer substantially increased in all vaccinated groups two weeks post-booster dose, and there was a significant difference when comparing G3 with G2 and G4 (*p*<0.05). We observed a decline in antibody titer at day-56 in all vaccinated chickens. However, titer remained above the baseline in G1, G2, and G3. Expectedly, no antibody response was observed in all pre-vaccination samples and unvaccinated controls (**Figure 2**).

The dynamic of antibody response within groups G1, G2, and G3 is similar in such a way that IgG titer was significantly higher on days 21, 35, and 56 than the baseline (day-0) with no difference among each day (**Figure 2**).

#### Mucosal antibody response

**Figure 3:**
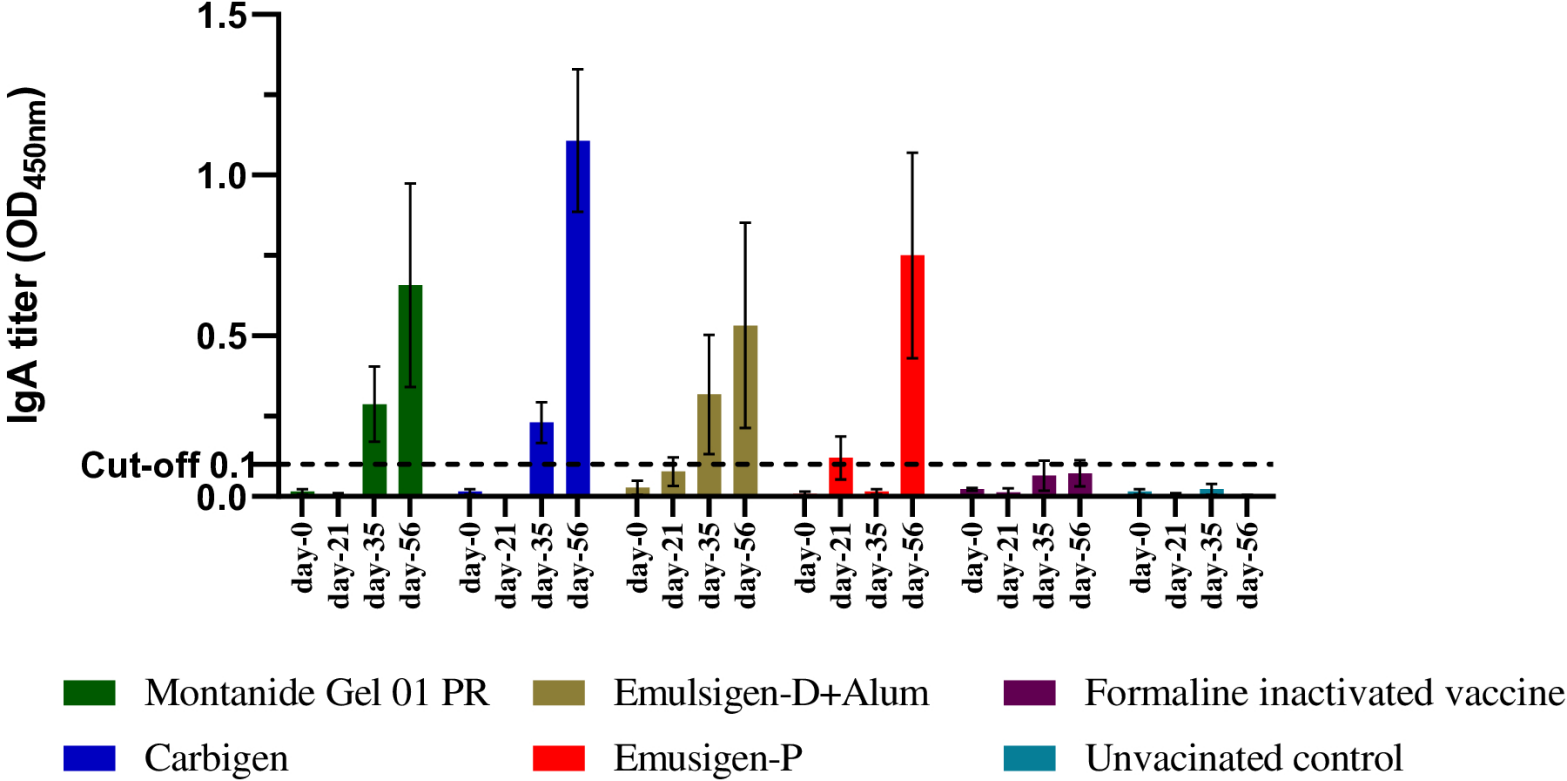
Mucosal IgA response to the candidate vaccines at days 0, 21, 35, and 56. Mucosal IgA titer was analyzed from tracheal and crop lavage samples using sandwich ELISA at 450 nm. Four chickens from each group were euthanized on each sampling day. The mean ± SEM is shown for each group on each sampling day. A cut-off value of 0.1 was used according to the manufacturer's guide.

In this study, mucosal IgA was not detectable after the first dose of vaccine in all the groups. A slight increment in IgA was measurable after the 2^nd^ dose (day-35) in G1 and G-2. Interestingly, on day-56, IgA titer increased significantly in all groups except formalin-inactivated and unvaccinated chickens. IgA titer was significantly higher on day-56 in chickens injected with Carbigen^®^ adjuvanted vaccine compared to formalin-inactivated and control groups (*p*<0.05) (**Figure 3**).

### Cellular immune response

#### Isolation and culturing of PBMCs

**Figure 4:**
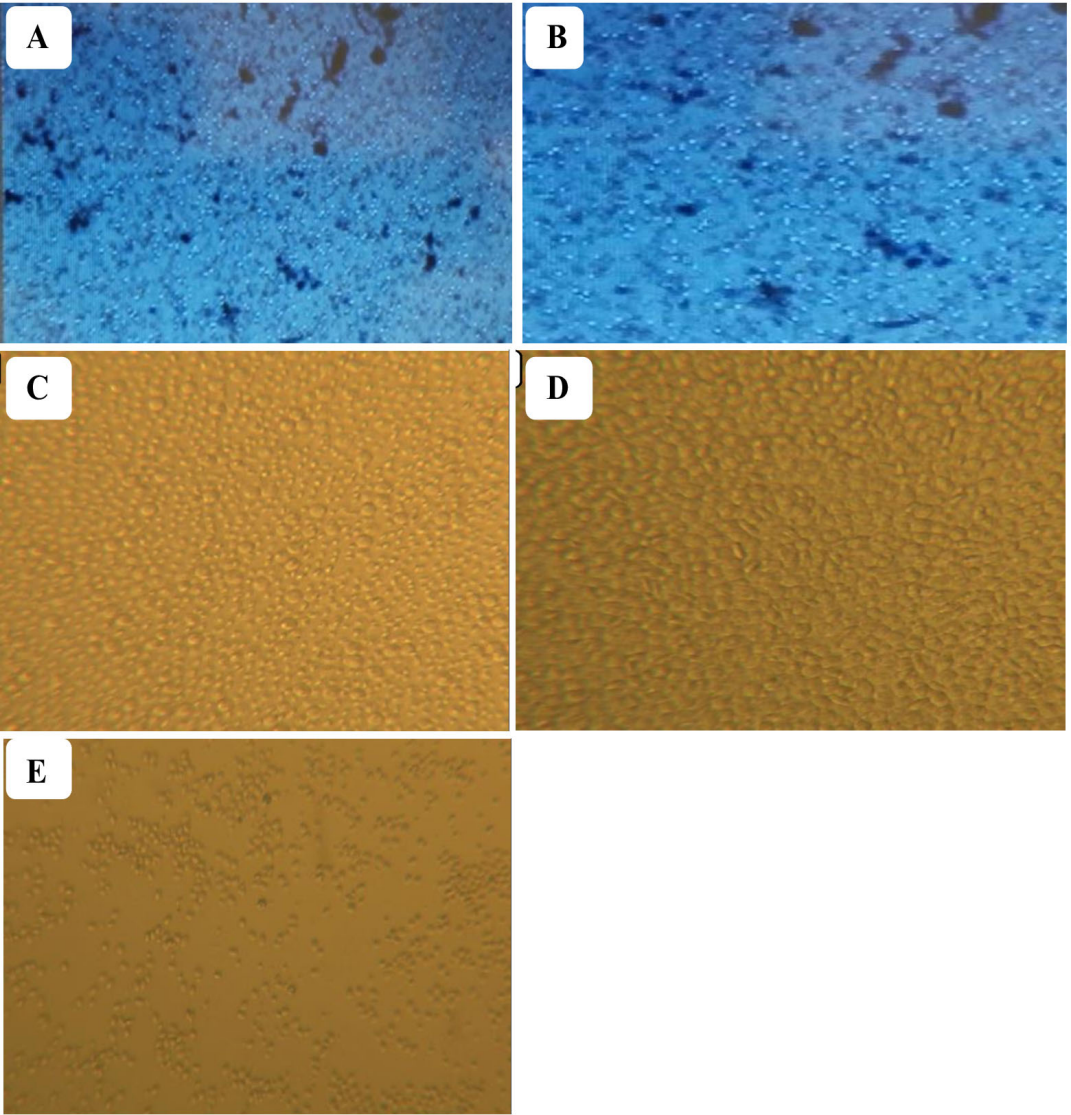
Isolated PBMCs from vaccinated **(A)** and non-vaccinated chickens **(B)** were pooled and stimulated with gamma-irradiated *P. multocida* antigen. Lymphocyte activation cocktail (LAC) was used to induce proliferation and served as a positive control. PBMCs treated with LAC **(C)** and PBMCs from vaccinated chickens treated with gamma irradiated *P. multocida* antigen **(D)** exhibited a significant proliferation as compared to unvaccinated **(E)** controls indicating vaccination-induced antigen specific priming.

PBMCs from vaccinated chickens responded to stimulation with gamma-irradiated *P. multocida* antigen with notable increase in size and number while PBMCs from unvaccinated chickens lack any detectable response. Similar proliferative response was observed in PBMCs stimulated with LAC. Collectively, these results indicate that gamma-irradiated vaccines successfully primed antigen specific immune cells (**Figure 4**).

#### Cytokine response

**Figure 5:**
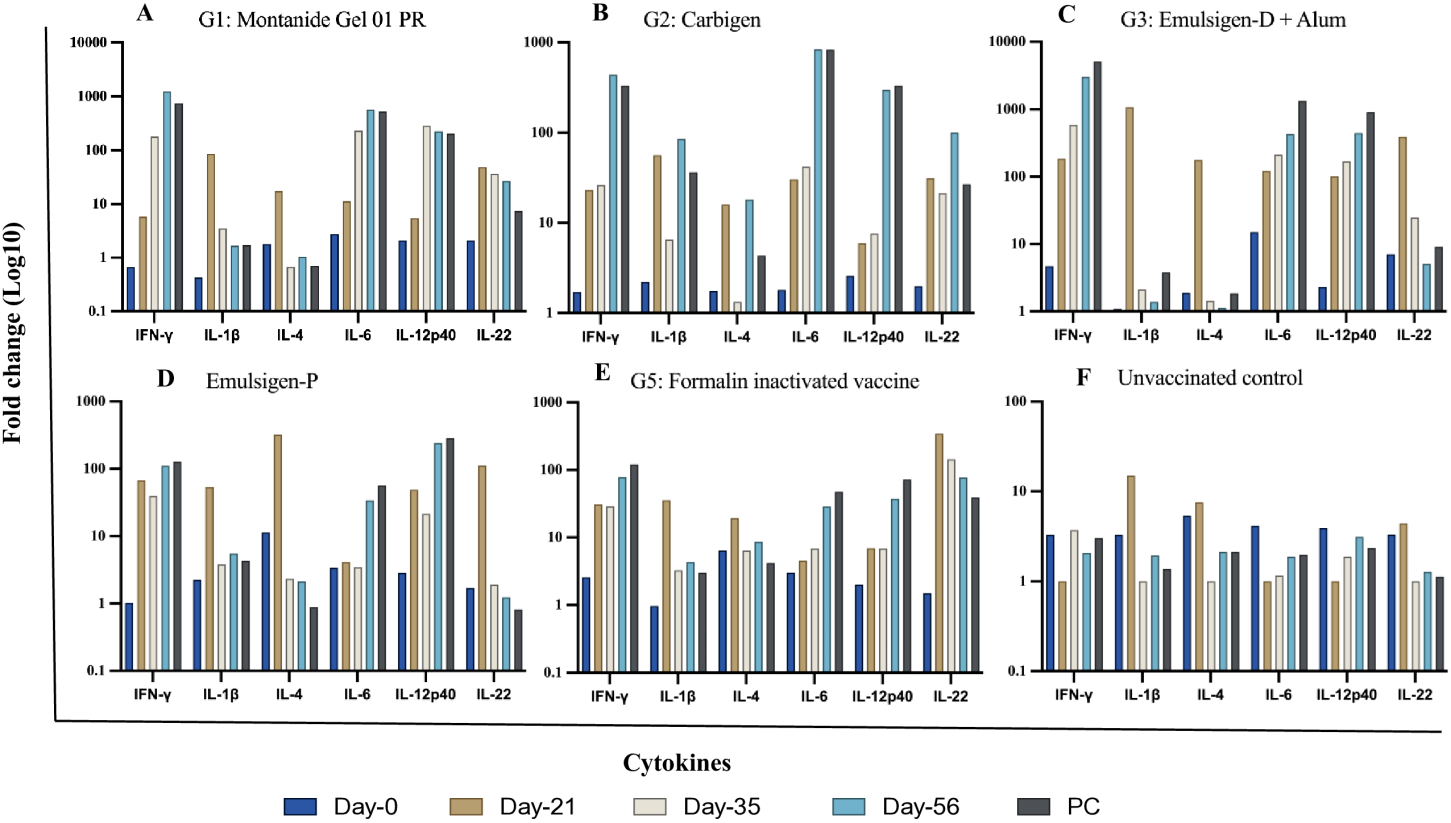
Cytokine gene expression analyses using RT-qPCR. Chickens were classified into 6 groups as Montanide Gel **(A)**, Carbigen **(B)**, Emulsigen-D+Alum **(C)**, Emulsigen-P **(D)**, Formalin inactivated vaccine **(E)**, Unvaccinated control **(F)**. PBMCs were analysed at days 0, 21, 35, 56 and seven days after challenge (AC). Relative fold change was initially normalized to the expression of the reference gene glyceraldehyde-3- phosphate dehydrogenase (GAPDH) and subsequently expressed as a fold change relative to expression levels of control group. Fold change was calculated using the 2^-AACt^ method.

**Figure 6:**
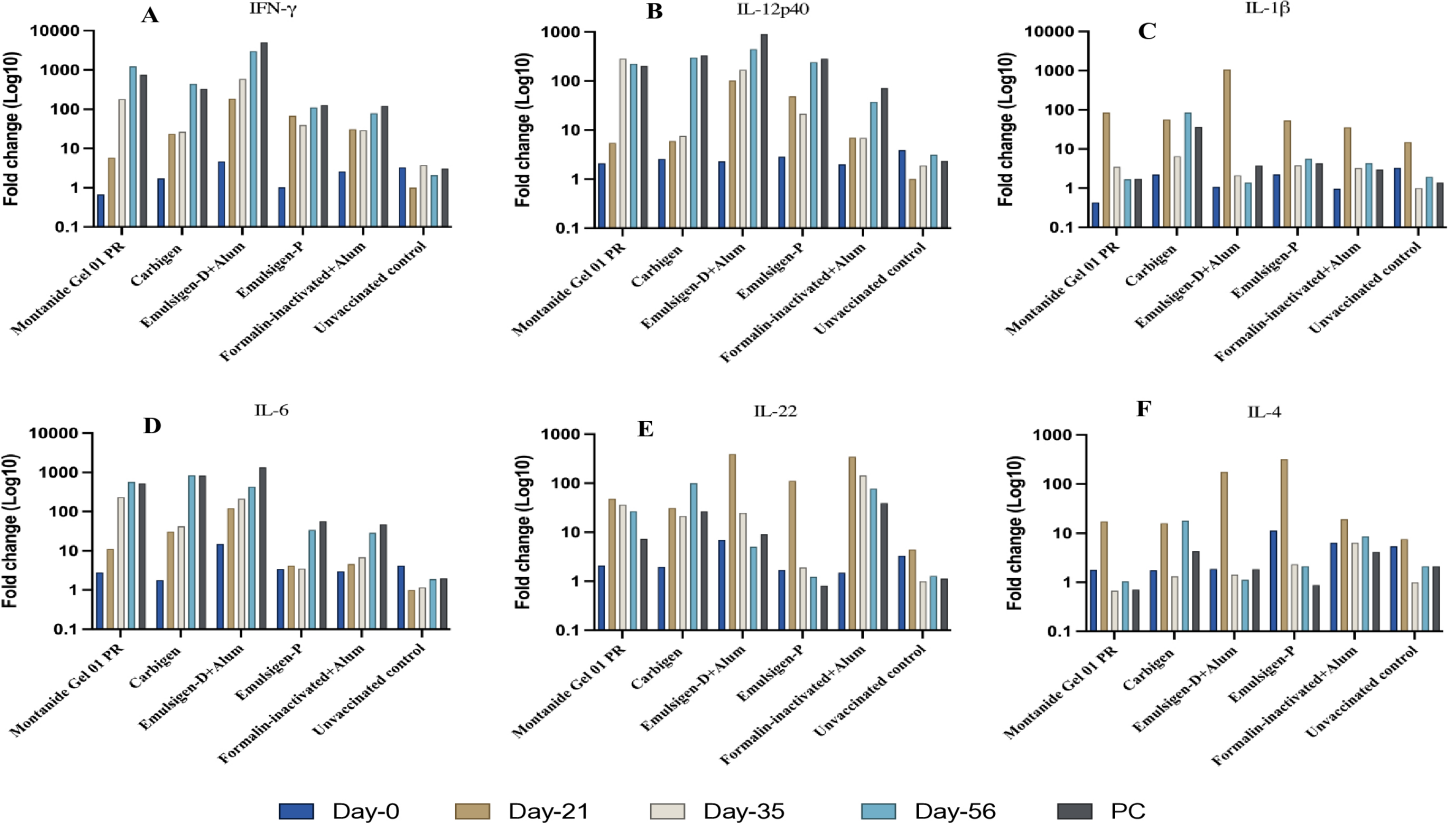
Cytokine gene expression analyses using RT-qPCR. IFN-γ **(A)**, IL-12p40 **(B)**, IL-1β **(C)**, IL-6 **(D)**, IL-22 **(E)**, IL-4 **(F)** in chicken PBMC at days 0, 21, 35, 56 and seven days after challenge (AC). Relative fold change was initially normalized to the expression of the reference gene glyceraldehyde-3-phosphate dehydrogenase (GAPDH) and subsequently expressed as a fold change relative to expression levels of control group. Fold change was calculated using the 2^-ΔΔCt^ method.

In this study, the expression of relevant cytokines was assessed, and there was a variable upregulation of cytokines across all the vaccinated groups. In G-1, IFN-γ, IL-6, and IL-12p40 showed increasing fold changes (FC) from day-21 through day-56 post vaccination; 6 to 1234, 11 to 568, and 6 to 224 FC respectively. On the other hand, IL-1β and IL-4 level remained indifferent at day-56 compared to the baseline. The expression of IL-22 did not change to the extent of IFN-γ, IL-6, and IL-12p40 even though it increased by 26-fold on day-56 (**Figures 5**, **6**).

In G-2, like G-1, IFN-γ, IL-6, and IL-12p40 had the highest expression level with fold changes of 440, 838, and 300 respectively at day-56. IL-22 level in this group increased by 100-fold at day-56 compared to baseline. This is contrary to G-3, where IL-22 level did not change during the study. However, IFN-γ (FC: 3019), IL-6 (FC: 430), and IL-12p40 (FC: 445) exhibited notable increment in expression level. IL-1β and IL-4 level dropped down to basal level despite an initial peak at day-21 post-vaccination.

The magnitude of change in cytokine expression in G-4 was not as extreme as the previous 3 groups, except for FC of 110 and 242 for IFN-γ and IL-12p40 respectively. Interestingly, in G-5, where chickens were vaccinated with formalin inactivated vaccine, cytokine response was not affected significantly except for IFN-γ (FC: 78) and IL-22 (FC: 77) on day-56 (**Figures 5**, **6**) Evaluation of efficacy of formulated vaccines

**Figure 7:**
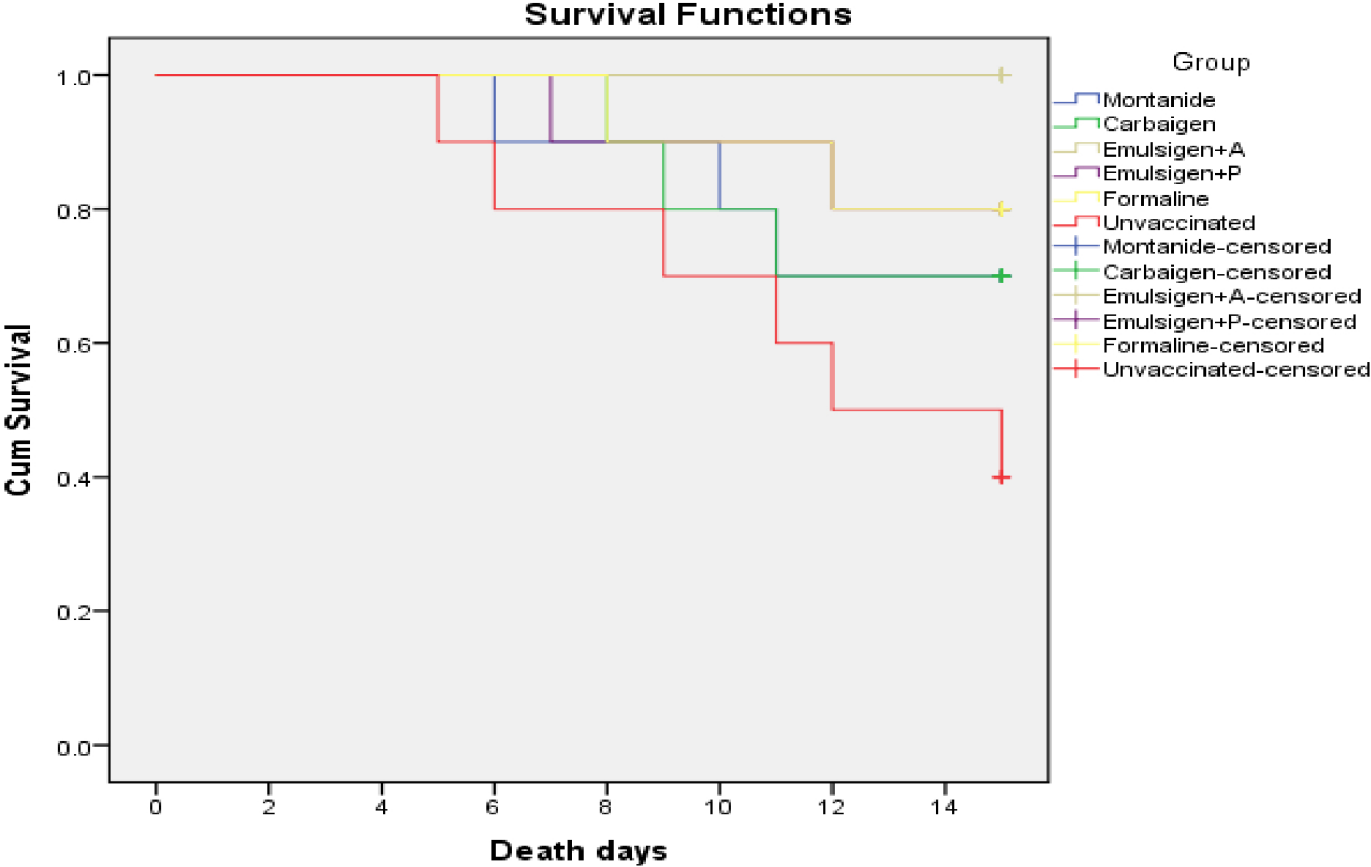
Survival curve analysis of the occurrence of death of chickens in each experimental group: each group comprises 10 chickens that had been followed for the period of 14 days. The chickens in the vaccinated and control groups were given 0.5 ml of avian *P. multocida* biotype A. The data were used to determine the kaplan-meier estimates (the product limit estimate) of both the control and the vaccinated groups.

The protective efficacy of the candidate vaccines was estimated in infection challenge study. A total volume of 0.5 ml bacterial suspension containing 2.5x10^7^ CFU/ml of avian *P. multocida* biotype A was administered to each individual experimental chicken and followed up for 14 days. Survival analysis is shown **Figure 7**. Vaccination with gamma-irradiated vaccine containing Emulsigen^®^-D with alum provided a complete protection against intramuscular challenge while vaccines adjuvanted with Montanide Gel 01 PR, Carbigen, Emulsigen-P and formalin inactivated FC vaccines had 50%, 50%, 66.7%, and 66.7% efficacy against the challenge, respectively. Clinical signs such as lameness, diarrhea, and death were observed in challenged chickens in a varying frequency indicated in **Table 2**.

**Table 2:** The occurrence of diarrhea, lameness and death across each experimental group.

Group	Total	Diarrhea (%)	Lameness (%)	Death (%)
Montanide Gel 01 PR	10	4 (40)	2 (20)	3 (30)
Carbigen	10	4 (40)	2 (20)	3 (30)
Emulsigen-D+Alum	10	0 (0)	0 (0)	0 (0)
Emulsigen-P	10	1 (10)	0 (0)	2 (20)
Formalin inactivated	10	4 (40)	4 (40)	2 (20)
Unvaccinated control	10	6 (60)	8 (80)	6 (60)
Overall	60	19 (31.7)	16 (26.7)	16 (26.7)

### Recovery of *P. multocida* after challenge

**Figure 8:**
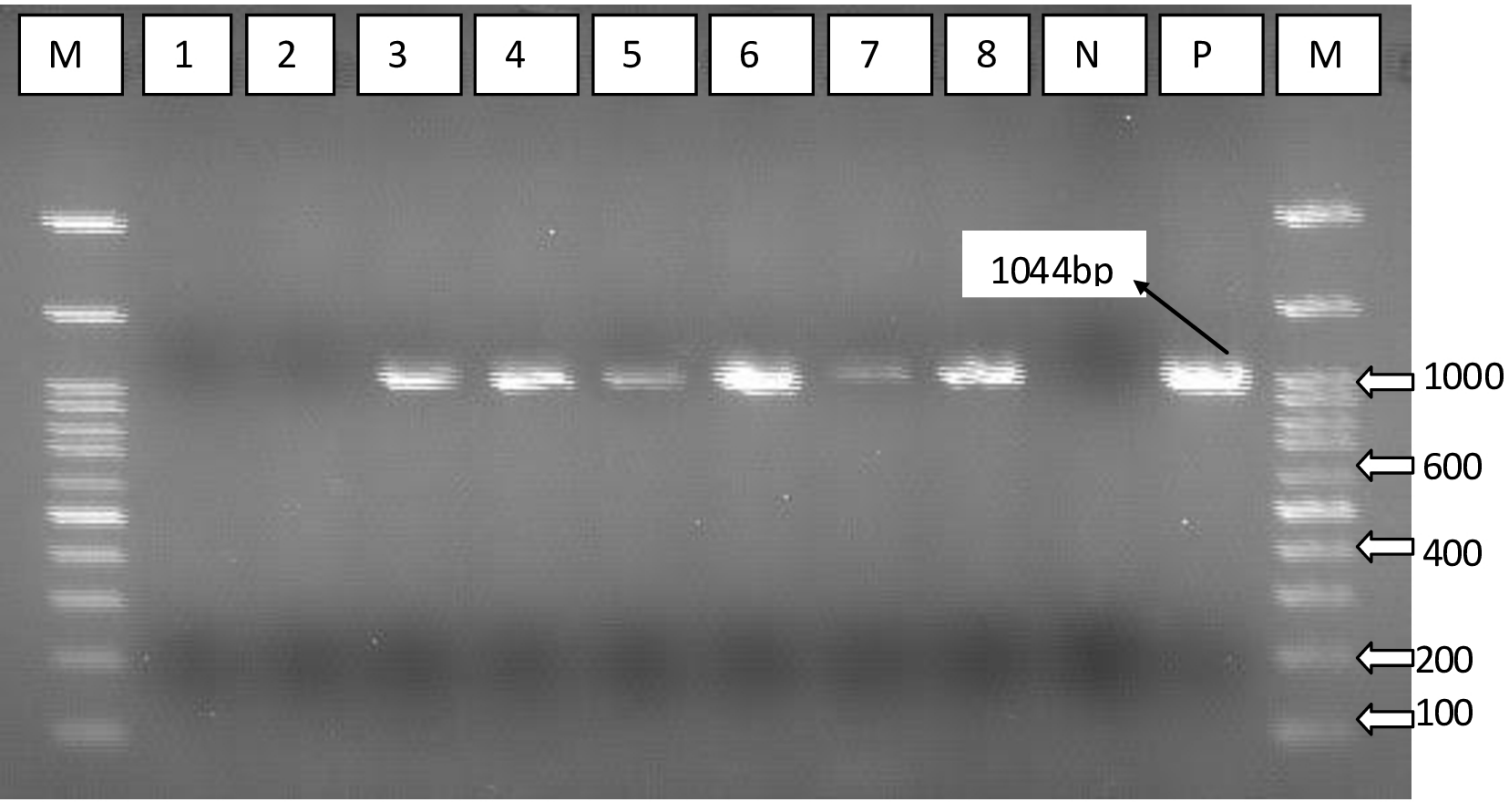
PCR using specific primer targeting capsular biosynthesis gene (capA). Lane 1, vaccine with Emulsigen®-D with aluminum hydroxide gel (Alum); lane 2, Montanide/01 PR gel; lane 3, Emulsigen®- P; lane 4, Carbigen®; lane 5, formalin killed vaccine; lane 6, unvaccinated control; lanes 7 and 8, *P. multocida* master seed strain; M, Molecular marker; N, Negative control; and P, positive control.

Following challenge, samples were collected randomly from chickens’ liver and lung tissue from each experimental group and analyzed using PCR to detect the presence of *P. multocida* capsular serotype A (capA) gene. Results demonstrated that the represented sample vaccination with both Emulsigen-D with aluminum hydroxide gel (Alum) and Montanide/01 PR gel adjuvanted was protected against *P. multocida* infection. Samples from these vaccinated groups tested negative for the capA gene, indicating a lack of detectable *P. multocida* capsular serotype A in the liver and lung tissues. Conversely, samples from all non-vaccinated groups tested positive for the capA gene, suggesting the presence of *P. multocida* capsular serotype A in these chickens following challenge (**Figure 8**).

## Discussion

In this study gamma-irradiated FC vaccines containing different adjuvants were formulated and evaluated for their safety, immunogenicity, and efficacy in chickens. IgG is the most prevalent immunoglobulin type in chicken sera, while secretory IgA is essential for mucosal immunity and is produced locally by plasma cells that are found at mucosal surfaces (35, 36). Thus, our assessment of systemic and mucosal immunity was based on serum IgG and secretory IgA respectively.

Accordingly, chickens vaccinated with Emulsigen-D+Alum seroconverted with a high IgG titer as compared to Carbigen and Emulsigen-P groups *(p*<0.05). This might possibly be due to the combination effect of the two adjuvants, Emulsigen^®^-D and Alum, as multi-adjuvanted vaccines can stimulate broad and robust protective immune responses by activating a variety of immune mechanisms required to fight infectious diseases (37, 38).

In addition, we observed that antibody titer persisted in groups receiving Montanide Gel 01 PR, Carbigen, and Emulsigen-D+Alum, but not in formalin inactivated vaccine group, at day-56 post primary vaccination. This finding aligns with previous reports that demonstrated oil-based adjuvants to induce a much more durable immune response than alum (39, 40). It has been indicated that emulsions have the ability to form depots that release antigens gradually generating a sustained stimulus to the immune system (41). Another suggested mechanism is through induction of apoptosis of cells which are subsequently phagocytosed by DCs, which get activated as a result (42). The decision to choose adjuvants needs to consider the cost and availability of the adjuvant as well as the conferred protection rather than mere immunogenicity parameters unless those parameters are known to correlate with protection.

On the other hand, there was no significant difference between the mean average antibody titer of chickens vaccinated with the vaccines containing Carbigen^®^, Montanide/01 PR gel, and formalin inactivated FC vaccine. This is contrary to Dessalegn et al. (9) who reported that Montanide/01 PR gel induced a higher antibody titer than formalin killed FC vaccine. This could be due to the additional third booster dose included in their study.

It is known that the avian *P. multocida* infects poultry species by the mucosal surface of the upper respiratory tract. Mucosal vaccines are attractive and efficient due to their ability to induce both systemic and local immunity, the latter providing immediate and effective response upon entry of the infectious agent (24). When administered via the intra nasal (IN) route, mucosal vaccines imitate the natural infection pathway of mucosal pathogens as avian *P. multocida*, perhaps eliciting a more protective immune response than injectable formulations (43).

In this study, all the gamma-irradiated preparations induced a detectable IgA without a significant difference in titer in between them. However, the formalin-inactivated vaccine failed to induce an IgA response. This can be explained by the variation in the route of vaccine administration i.e. all the gamma-irradiated vaccines were administered via mucosal routes while the formalin-inactivated vaccine was injected intramuscularly. Mucosal vaccination, but not parenteral vaccines, are known to induce both systemic and local immunity due to stimulation of B and T cells that migrate to systemic secondary tissue as well as different mucosal compartments (44).

In addition to humoral immune response, this study evaluated cellular immune response, which also plays an inevitable role in defense against FC. The PBMC compartment was investigated for this purpose i.e. PBMC proliferation and cytokine gene expression in response to vaccination were the endpoints for the cellular immunity. PBMCs were isolated and cultured in the presence of *P. multocida*, to mimic a repeat exposure, and only post-vaccination PBMC proliferated notably in response to the re-stimulation. This response was evident by the observed increase in size, granularity, and overall number of PBMCs compared to unvaccinated PBMCs.

This response can be ascribed to the already primed population of PBMCs in vaccinated chickens having a lower threshold for re-activation by the same antigen (*P. multocida*), thereby exhibiting a prompt response. The fact that vaccinated PBMCs respond to re-stimulation with *P. multocida* antigen is indicative of activation of specific immune response due to vaccination.

Cytokines are crucial in orchestrating defense against infection and vaccination (45), and thus the dynamic of their expression level can be used as probe to study immune responses generated in context of infection and vaccination. Thus, this study assessed the cytokine response of chickens to vaccination at the level of mRNA transcript. The gene expression profile of a panel of cytokines, such as IFN-γ, IL-12p40, IL-4, IL-22, IL-1β, and IL-6 was studied.

IFN-γ and IL-12p40 transcripts were upregulated by hundreds to thousands folds in all vaccinated chickens as compared to unvaccinated group. On the other hand, IL-4 expression was not affected due to vaccination except for the subtle (compared to IFN-γ and IL-12p40) spikes observed at day-21 post-vaccination which subsequently declined back to baseline in all the vaccinated groups. The local cytokine milieu is an important factor in governing the type of T cell effector response that is induced (46). IFN-γ and IL-4 are known for their mutually antagonistic functions (47, 48). IL-12 and IFN-γ potently induce type 1 immune responses and IL-4 and is important for the induction of type 2 immune responses (49). IL-12 induces INF-γ synthesis and has a proliferative effect on chicken splenocytes. A variety of immune cells such as NK cells and Th1 cells produce IFN-γ in response to IL-12 from macrophages. IFN-γ in turn activates macrophages and boosts cytotoxic T cells, allowing them to eliminate intracellular parasites and infected cells (50, 51). Based on our data, it can be stated that the gamma-irradiated vaccines induced a predominantly Th1 response which is beneficial against intracellular pathogens (52, 53).

As mentioned before, there was a slight upregulation of the Th2 cytokine, IL-4, mRNA transcripts in vaccinated chickens. It has been reported that Th2 immune response helps to counterbalance damages induced by an elevated Th1 mediated inflammation (54). Other studies have also highlighted Th1/Th2 imbalance as a mechanism for pathologies observed following infection or chemical damage (55, 56)

In our study, cytokine mRNA expression and thus cellular response to formalin inactivated vaccine was of a lesser magnitude relative to gamma-irradiated vaccines as observed from the fold changes. Similar finding was reported by Sedeh et al. (57) for gamma-irradiated avian influenza vaccine. Nevertheless, the fold change for IL-22 was relatively higher in the group that received the formalin treated vaccine. Being secreted by a wide range of immune cells, IL-22 has been reported to limit Th1 responses and promotes regulatory T cells that inhibit the immune system and cytokines production (58). This is in accordance with our observation of the relatively lower responses of IL-12 and IFN-γ in this group.

The other cytokines involved in this study were IL-1β and IL-6. The level of IL-1β transcript increased at day-21 but could not persist in all vaccinated groups. Whereas IL-6 expression persisted throughout the study period in groups—Montanide Gel 01 PR, Carbigen, and Emulsigen-D+alum. Both cytokines are highly proinflammatory and are critical for initiating an acute-phase immune response against invading pathogens and triggering a variety of immune cells, such as T cells and macrophages (59, 60).

Lastly, the efficacy (protection from death) of the formulated vaccines ranged from 50% (Montanide Gel 01 PR and Carbigen group) to 100% (Emulsigen-D+alum group). The vaccines used in G-4 (Montanide-P) and G-5 (formalin inactivated) showed an efficacy of 66.7%. In light of the data generated in this study, we can conclude that gamma irradiation offers an effective alternative to produce safe and efficacious mucosal vaccines against fowl cholera with the potential to induce a broad range of humoral (systemic and local) as well as cellular immunity. Emulsigen-D+alum performed better in terms of immunogenicity and efficacy and needs to be backed up by further studies involving large sample size, various routes, doses and formulation. As a limitation, the sample size used for assessing the mucosal immunity was small, thus it might not enable detection of small effect size. In addition, vaccine efficacy was only tested against one bacterial strain. Furthermore, we could not investigate gamma-irradiated formulations adjuvanted with only alum and formalin inactivated preparation adjuvanted with emulsions due to budget and time constraints and could be addressed in future studies.

## Data Availability

The original contributions presented in the study are included in the article/supplementary material. Further inquiries can be directed to the corresponding authors.
